# Development and validation of a prognostic nomogram for predicting cancer-specific survival in lymph node-negative elderly esophageal cancer patients: A SEER-based study

**DOI:** 10.1097/MD.0000000000034441

**Published:** 2023-07-28

**Authors:** Lang Qin, Lianlian Chen, Xiaowei Tie, Xinwei Guo, Faming Yang, Yangchen Liu

**Affiliations:** a Department of Radiotherapy, Taixing Clinical College of Bengbu Medical College, Bengbu, China; b Department of Oncology, The First Affiliated Hospital of Anhui University of Science and Technology, Huainan, China; c Department of Gastroenterology, Taixing Clinical College of Bengbu Medical College, Bengbu, China.

**Keywords:** cancer-specific survival, elderly patient, esophageal cancer, nomogram, SEER

## Abstract

In this study, we explored the prognostic risk factors of elderly patients (≥65 years old) with lymph node-negative esophageal cancer (EC) and established a nomogram to evaluate the cancer-specific survival of patients. The surveillance, epidemiology, and end results database was used to collect data on patients diagnosed with EC. Univariate and multivariate Cox analyses were used to determine independent prognostic factors, and the nomogram for predicting cancer-specific survival of EC patients was constructed based on the independent prognostic factors obtained from the multivariate Cox analysis. To evaluate the predictive ability of the nomogram, calibration curves, concordance index (C-index), receiver operating characteristic curves, and decision curve analysis were conducted. Kaplan–Meier method was used to analyze the long-term outcomes of EC patients with different risk stratifications. A total of 3050 cases with lymph node-negative EC were randomized into the training cohort (1525) and the validation cohort (1525). Cancer-specific mortality at 1, 3, and 5 years in the entire cohort was 30.7%, 41.8%, and 59.2%, respectively. In multivariate Cox analysis, age (*P* < .001), marital status (*P* < .001), tumor size (*P* < .001), Tumor-node-metastasis stage (*P* < .001), chemotherapy (*P* = .011), radiotherapy (*P* < .001), and surgery (*P* < .001) were independent prognostic factors. The C-index for the training cohort was 0.740 (95% confidence interval [CI]: 0.722–0.758), and the C-index for the validation cohort was 0.738 (95% CI: 0.722–0.754). The calibration curve demonstrated the great calibration ability of the nomogram. Based on the area under the receiver operating characteristic curve, the nomogram demonstrated a higher sensitivity than the tumor-node-metastasis stage. Decision curve analysis showed the good clinical utility of the nomogram. The risk stratification system was established using the Kaplan–Meier curve and verified by the log-rank test (*P* < .001). The nomogram and risk stratification system can improve the accuracy of prediction to help clinicians identify high-risk patients and make treatment decisions.

## 1. Introduction

Esophageal cancer (EC) is one of the leading causes of cancer death worldwide, with the seventh incidence rate and the sixth mortality rate, which means that 1 in 18 patients dying from cancer in 2020 is caused by EC, as the incidence rises in parallel with the average age of the global population, more elderly individuals will be diagnosed with EC in the future.^[1–3]^ Numerous studies have also shown that older age is an important risk factor for death in EC patients.^[4,5]^ Therefore, further attention should be paid to the prognosis of the elderly.

According to the National Comprehensive Cancer Network guidelines, surgery-based comprehensive treatment is the mainstream treatment option for lymph node-negative EC. However, for the elderly, it is significant to assess not only the patient tolerance but also the ability to recover from surgical trauma. Surgery is not the first choice for the part of elderly patients who cannot be successfully operated on due to poor health, and adjuvant therapy is still optional for lymph node-negative EC patients. The relationship between adjuvant therapy and the prognosis of node-negative EC patients has been explored in numerous studies, but the conclusions remain uncertain.^[6–10]^ Furthermore, prognostic factors such as age, sex, and tumor size should be considered when evaluating these patients. Therefore, we aim to achieve a more accurate prognosis analysis by analyzing information on demographic characteristics, clinicopathological characteristics, and treatment methods in the surveillance, epidemiology, and end results Program (SEER) databases.

The tumor-node-metastasis (TNM) stage system is widely recognized and applied in tumor prognosis. However, in recent years, nomograms have shown a better accuracy in the prediction of prognosis than the TNM stage in various types of cancer.^[11–14]^ Although there have been some nomograms in the EC field,^[15–17]^ there have been no relevant studies on node-negative elderly patients with EC. Based on the SEER database, we developed and validated a nomogram that can be used to predict cancer-specific survival (CSS) at 1, 3, and 5 years in node-negative elderly EC patients.

## 2. Methods

### 2.1. Patient selection

**Figure 1.**
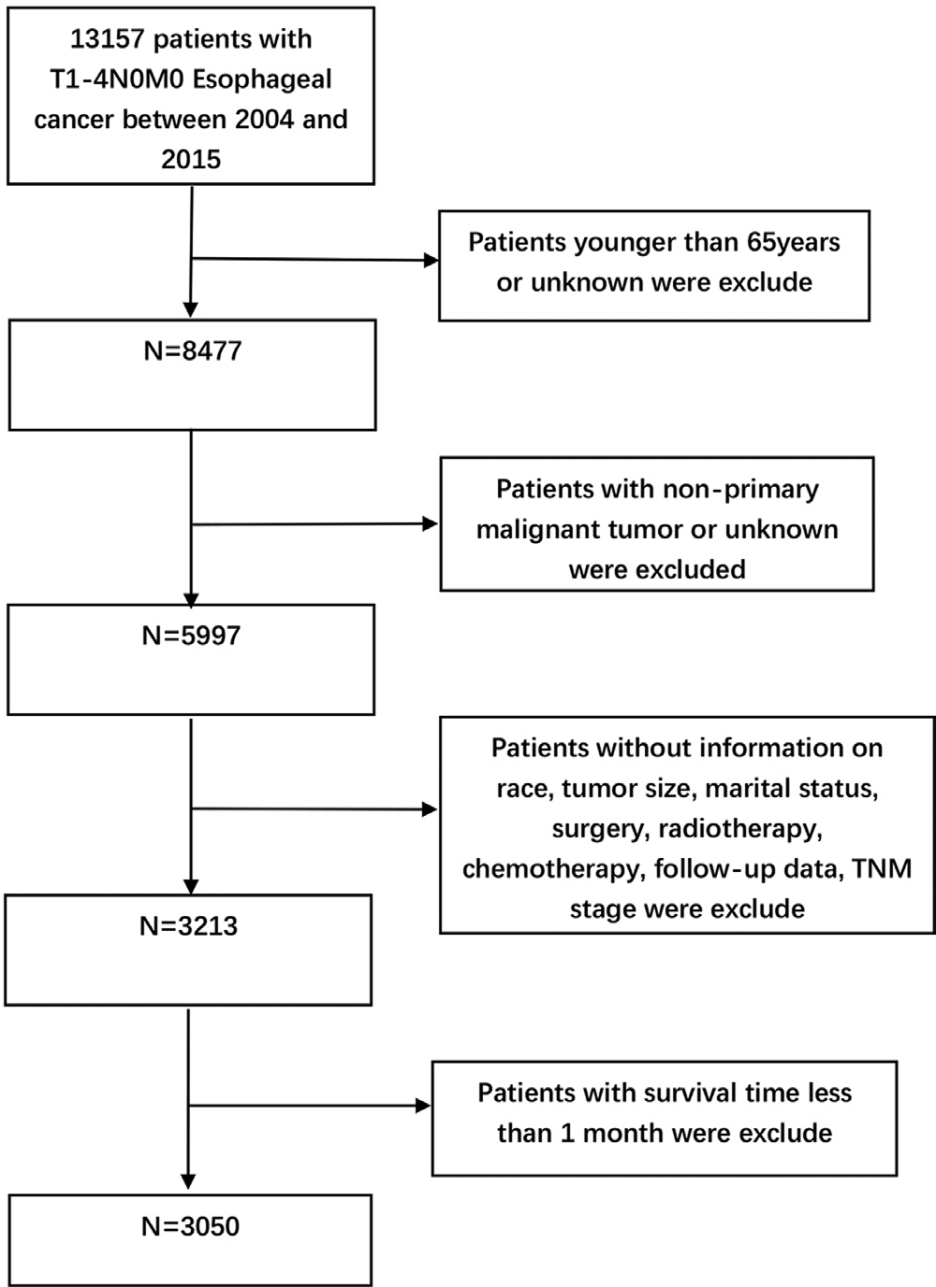
General flowchart of this study.

We retrieved data online using the SEER*Stat software Version 8.4.0.1 (http://seer.cancer.gov/seerstat/). In our study, a total of 13,157 patients with T1-4N0M0 stage EC between 2004 and 2015 were retrieved from the “incidence-SEER Research Plus Data, 18 Registries” database. The exclusion criteria were: Patients younger than 65 years old or with unknown age; Non-primary malignant tumors or patients with unknown tumor status; Patients with no information on race, tumor size, marital status, surgery, radiotherapy, chemotherapy, follow-up data, and TNM stage; Patients with a survival time of <1 month. A total of 3050 patients were included in the study through the above process (Fig. 1). A 5:5 ratio was randomly divided between the training cohort (n = 1525) and the validation cohort (n = 1525).In this study, patient demographic characteristics, including age, sex, race, and marital status; clinicopathological characteristics, including primary tumor site, histology, tumor size, grade, and TNM stage; treatment information, including surgery, radiotherapy, and chemotherapy; and follow-up data, including survival time and survival status were collected through the SERR database. The observational endpoint of the study was CSS, the time between diagnosis and death attributable to EC. Moreover, since age and tumor size are discrete type variables, the optimal cutoff value in the prognostic analysis of EC patients was judged using X-tile (Yale University, version 3.6.1) and thus transformed into categorical variables.

### 2.2. Statistical analysis

Pearson chi-square test was used to analyze the differences in the distribution of categorical variables between the training cohort and the validation cohort. The univariate Cox analysis was used to select the statistically significant variables to include in the multivariate Cox analysis, and the nomogram was established based on the independent prognostic factors obtained from the multivariate Cox analysis. For the validation of the nomogram, the calibration curve was used to evaluate calibration ability, concordance index (C-index) was performed to estimate discrimination ability, receiver operating characteristic (ROC) curve was used to compare the nomogram and the TNM stage system differences in recognition ability. The clinical application was evaluated using decision curve analysis (DCA). To decrease the overfit bias, bootstraps with 1000 resamples were used.

All the statistical analyses of this study were performed in the R (version 4.2.1, http://www.R-project.org). The “tableone” package was used for the chi-square test. The “rio” package was used for data output. The “survival” package and “survminer” package were used for the Kaplan–Meier curve and the log-rank test. The “survival” package, “forestplot” package, and “survminer” package were used for univariate and multivariate Cox analysis, nomogram establishment, and C index calculation. The “rms” package and “survival” package were used for the calibration curve establishment. The “riskRegression” package and “survival” package were used to establish the ROC curve. The “rms” package, “ggDCA” package, “ggprism” package, and “survival” package were used to establish the DCA. *P* < .05 was considered statistically different.

### 2.3. Prognostic risk stratification

The scores obtained from the nomogram were assigned to each independent prognostic factor and the total score was calculated by adding up the scores of each prognostic variable. The best cutoff value of the total score was determined using X-tile and was divided into 3 categories (low, middle, and high-risk groups). The Kaplan–Meier survival curve and the log-rank test were used to analyze the differences in long-term prognosis for CSS of the 3 subgroups.

### 2.4. Ethical Statement

Publicly available data was obtained from the SEER database (http://seer.cancer.gov/seerstat/) for use in the current study. Therefore, the study exempted institutional review board approval.

## 3. Results

### 3.1. Patient characteristics

A total of 3050 patients were enrolled from the SEER database for the analysis, including 1525 patients in the training cohort and 1525 patients in the validation cohort. The demographic, clinicopathological, and treatment information were summarized (Table 1). In the entire cohort, 73.44% (2240/3050) of the patients were male, 87.02% (2654/3050) of the patients were white, 56.98% (1783/3050) of the patients were adenocarcinoma, 48.89% (1479/3050) were stage I, 43.41% (1324/3050) were stage II, 45.31% (1382/3050) of patients underwent surgery, while 56.95% (1737/3050) and 51.70% (1557/3050) of patients underwent radiotherapy and chemotherapy, respectively. The median follow-up time for the entire study cohort was 23 months (range: 1–179 months), and a total of 2373 deaths occurred during the follow-up period, including 1753 cancer-specific deaths and 620 deaths from other causes. Cancer-specific mortality rates at 1, 3, and 5 years were 30.7%, 41.8%, and 59.2%, respectively.

**Table 1:** Characteristics of the training and validation cohorts (χ^2^ test).

Variables		Whole cohort n = 3050	Training cohort n = 1525	Validation cohort n = 1525	*P*
Age	65–74	1588 (52.07)	786 (51.54)	802 (52.59)	.701
	75–83	1048 (34.36)	535 (35.08)	513 (33.64)	
	>83	414 (13.57)	204 (13.38)	210 (13.77)	
Sex	Female	810 (26.56)	406 (26.62)	404 (26.49)	.967
	Male	2240 (73.44)	1119 (73.38)	1121 (73.51)	
Race	Black	242 (7.93)	120 (7.87)	122 (8.00)	.416
	White	2654 (87.02)	1320 (86.56)	1334 (87.48)	
	Others	154 (5.05)	85 (5.57)	69 (4.52)	
Marital status	Married	1808 (59.28)	891 (58.43)	917 (60.13)	.357
	Unmarried	1242 (40.72)	634 (41.57)	608 (39.87)	
Site	Upper	188 (6.16)	94 (6.16)	94 (6.16)	.244
	Middle	591 (19.38)	317 (20.79)	274 (17.97)	
	Lower	1838 (60.26)	906 (59.41)	932 (61.11)	
	Others	433 (14.20)	208 (13.64)	225 (14.75)	
Histology	EAC	1738 (56.98)	874 (57.31)	864 (56.66)	.685
	ESCC	1067 (34.98)	535 (35.08)	532 (34.89)	
	Others	245 (8.03)	116 (7.61)	129 (8.46)	
Grade	Well	235 (7.70)	109 (7.15)	126 (8.26)	
	Moderately	1237 (40.56)	621 (40.72)	616 (40.39)	.701
	Poorly	1063 (34.85)	528 (34.62)	535 (35.08)	
	Undifferentiated	38 (1.25)	21 (1.38)	17 (1.11)	
	Unknown	477 (15.64)	246 (16.13)	231 (15.15)	
Size	≤22 mm	962 (31.54)	482 (31.61)	480 (31.48)	.704
	23–59 mm	1468 (48.13)	742 (48.66)	726 (47.61)	
	>59 mm	620 (20.33)	301 (19.74)	319 (20.92)	
Stage	I	1479 (48.49)	741 (48.59)	738 (48.39)	.682
	II	1324 (43.41)	667 (43.74)	657 (43.08)	
	III	247 (8.10)	117 (7.67)	130 (8.52)	
Surgery	No	1668 (54.69)	836 (54.82)	832 (54.56)	.913
	Yes	1382 (45.31)	689 (45.18)	693 (45.44)	
Radiation	No	1313 (43.05)	652 (42.75)	661 (43.34)	.770
	Yes	1737 (56.95)	873 (57.25)	864 (56.66)	
Chemotherapy	No	1473 (48.30)	727 (47.67)	746 (48.92)	.514
	Yes	1577 (51.70)	798 (52.33)	779 (51.08)	

EAC = esophageal adenocarcinoma, ESCC = esophageal squamous cell carcinoma, TNM = tumor-node-metastasis.

### 3.2. Univariate and multivariate Cox analysis

Univariate analysis showed that age (*P* < .001), marital status (*P* < .001), race (*P* = .001), primary site (*P* < .001), histology (*P* < .001), grade (*P* < .001), tumor size (*P* < .001), TNM stage (*P* < .001), surgery (*P* < .001), chemotherapy (*P* = .019), and radiotherapy (*P* < .001) were significantly associated with CSS and were included in the multivariate Cox regression model. In the multivariate analysis, age (*P* < .001), marital status (*P* < .001), tumor size (*P* < .001), TNM stage (*P* < .001), surgery (*P* < .001), chemotherapy (*P* = .004), and radiotherapy (*P* < .001) were independent prognostic factors for CSS (Table 2) and were included in the establishment of the nomogram.

**Table 2:** Univariate and multivariate Cox analysis of cancer-specific survival for esophageal cancer patients.

	Univariate analysis	*P*	Multivariate analysis HR (95% CI)	*P*
Age (yr)		<.001		
	65–74		Reference	
	75–83		1.317 (1.129–1.535)	<.001
	≥83		1.501 (1.264–1.783)	<.001
Sex		.001		
	Female		Reference	
	Male		1.050 (0.892–1.235)	.559
Marital status		<.001		
	Married		Reference	
	Unmarried		1.307 (1.132–1.508)	<.001
Race		.001		
	Black		Reference	
	White		0.908 (0.706–1.157)	.422
	Others		1.104 (0.764–1.597)	.598
Primary site		<.001		
	Lower		Reference	
	Middle		1.070 (0.880–1.300)	.500
	Upper		1.003 (0.752–1.338)	.983
	Others		1.143 (0.941–1.389)	.178
Histology		<.001		
	EAC		Reference	
	ESCC		1.077 (0.898–1.293)	.423
	Others		1.153 (0.899–1.478)	.262
Grade		<.001		
	Well		Reference	
	Moderately		1.203 (0.875–1.653)	.255
	Poorly		1.539 (1.112–2.130)	.009
	Undifferentiated		1.228 (0.635–2.372)	.542
	Unknown		1.170 (0.824–1.661)	.381
Size (mm)		<.001		
	>59		Reference	
	1–22		0.435 (0.352–0.538)	<.001
	23–59		0.761 (0.649–0.893)	<.001
Stage		<.001		
	I		Reference	
	II		1.091 (0.937–1.274)	.260
	III		1.690 (1.307–2.187)	<.001
Surgery		<.001		
	No		Reference	
	Yes		0.285 (0.236–0.349)	<.001
Chemotherapy		.019		
	No		Reference	
	Yes		0.755 (0.621–0.916)	.004
Radiotherapy		<0.001		
	No		Reference	
	Yes		0.601 (0.486–0.743)	<.001

CI = confidence interval, EAC = esophageal adenocarcinoma, ESCC = esophageal squamous cell carcinoma, HR = hazard ratio, TNM = tumor-node-metastasis.

### 3.3. Construction and validation of the nomogram

**Figure 2.**
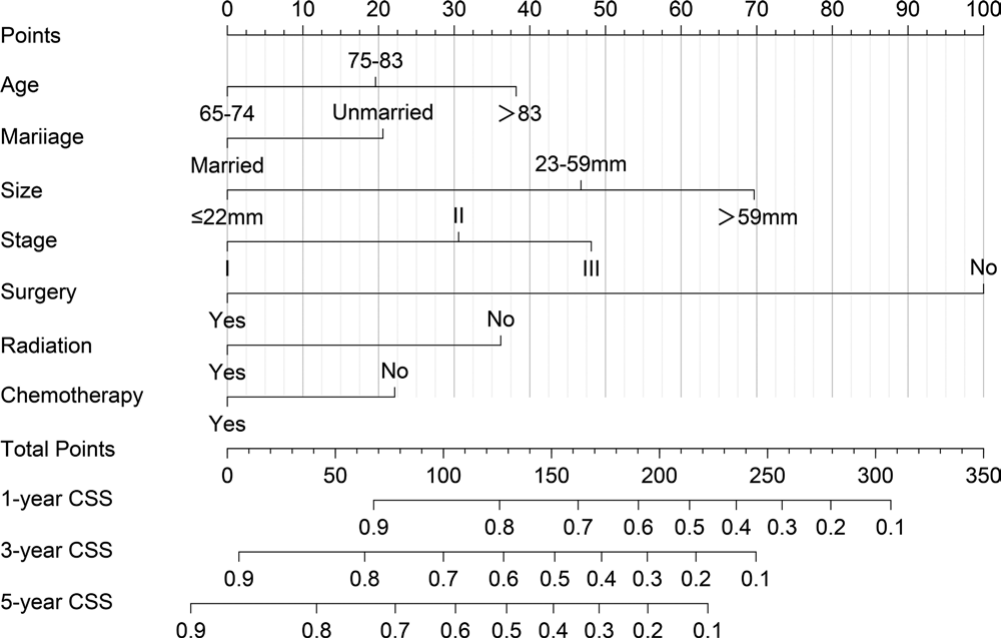
Nomogram for predicting 1-, 3- and 5-yr cancer-specific survival of patients with esophageal cancer.

**Figure 3.**
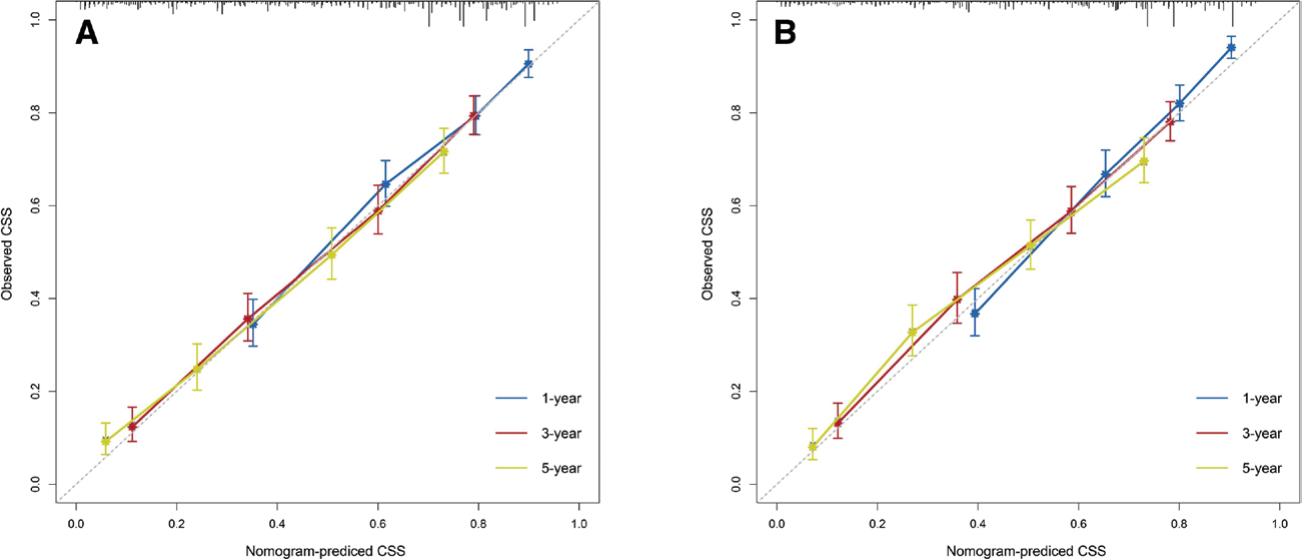
Calibration curves of the nomogram. (A) Calibration curves of 1-, 3- and 5-yr cancer-specific survival in the training cohort; (B) Calibration curves of 1-, 3- and 5-yr cancer-specific survival in the validation cohort.

**Figure 4.**
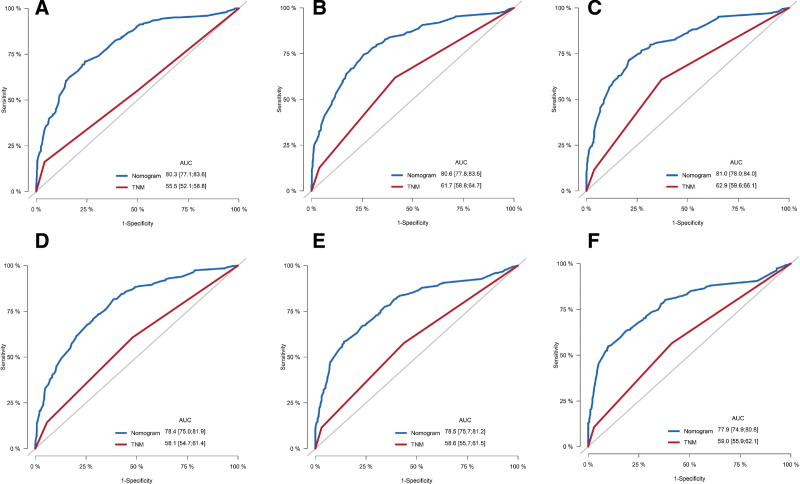
Receiver operating characteristic curves of the nomogram versus tumor-node-metastasis (TNM) stage. (A, B, C) For 1-, 3- and 5-yr cancer-specific survival in the training cohort; (D, E, F) for 1-, 3- and 5-yr cancer-specific survival in the validation cohort.

**Figure 5.**
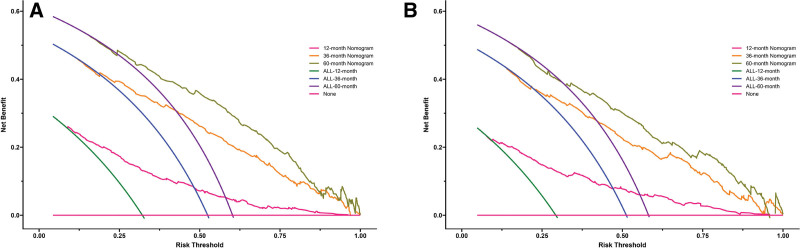
Decision curve analysis for survival prediction. (A) For 1-, 3-and 5-yr cancer-specific survival in the training cohort; (B) for 1-, 3- and 5-yr cancer-specific survival in the validation cohort.

Based on the results of the multivariate Cox analysis, we established a nomogram (Fig. 2). All the independent prognostic factors include age, marital status, tumor size, TNM stage, surgery, chemotherapy, and radiotherapy. In the training cohort, the C-index of the prognostic nomogram and the TNM stage were 0.740 (95% confidence interval [CI]: 0.722–0.758) and 0.574 (95%CI: 0.554–0.594), respectively. Meanwhile, in the validation cohort, the C-index of the prognostic nomogram and the TNM stage were 0.738 (95%CI: 0.722–0.754) and 0.574 (95%CI: 0.554–0.594), respectively. To verify the calibration capability, we summarized the calibration curves at 1-, 3-, and 5-year intervals for the nomogram (Fig. 3). The calibration curves of the nomogram for both the training cohort and the validation cohort indicate that the nomogram-predicted survival is highly consistent with the actual survival rate. The ROC curves proved that the nomogram established in this study had a higher prediction efficacy than the TNM stage at 1-, 3-, and 5- years (Fig. 4). The area under the ROC curves of the training cohort at 1-, 3-, and 5- years were 0.803 (95%CI: 0.771–0.836), 0.806 (95%CI: 0.778–0.835), 0.810 (95%CI: 0.780–0.840), respectively. The area under the ROC curves of the validation cohort at 1-, 3-, and 5- years were 0.784 (95%CI: 0.750–0.819), 0.785 (95%CI: 0.757–0.812), 0.779 (95%CI: 0.749–0.808), respectively. The DCA further indicated that the nomogram has great clinical applicability in predicting 1-, 3-, and 5-year survival (Fig. 5).

### 3.4. Establishment of risk stratification

**Figure 6.**
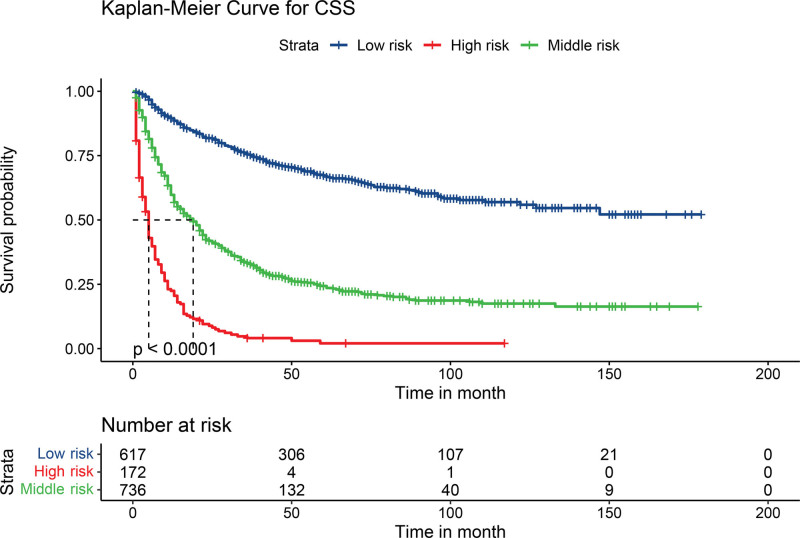
Kaplan–Meier method estimate of cancer-specific survival in the training cohort. Low risk refers to a total score of <135, high risk refers to a total score of >244, and middle risk is between 136 and 243.

The best cutoff value of the total score was calculated using X-tile software, and the patients were divided into 3 subgroups: low risk (<135 points), middle risk (136–243 points), and high risk (>244 points). We observed a significant survival difference among the 3 groups of patients according to the log-rank test (*P* < .001) (Fig. 6).

## 4. Discussion

In this study, we established a prognostic nomogram of CSS for predicting lymph node-negative elderly EC patients for 1, 3, and 5 years based on the SEER database, which performed excellently in the internal validation. Age, marital status, tumor size, TNM stage, surgery, chemotherapy, and radiotherapy were considered independent prognostic factors for CSS. However, since only the poorly differentiated patients were statistically significant in the results of the multivariate Cox regression (*P* = .009), we did not include tumor grade in the establishment of the nomogram. The C-index of both the training cohort and the validation cohort was >0.70, indicating that the nomogram constructed in this study had a satisfactory prediction. Furthermore, the ROC curve showed that the nomogram predicted more efficiently than the existing TNM stage system. We verified the good agreement between the predicted survival probability of the nomogram and the actual survival probability of the patients by using the calibration curves. To accurately evaluate the prognosis of patients, we assigned each independent prognostic variable a score by nomogram and calculated the total patient score. We divided EC patients into 3 groups according to different scores using X-tile, and the Kaplan–Meier curve showed huge survival differences. With the easy-to-use risk stratification system, clinicians can judge the prognosis of EC patients and take treatment or follow-up measures accordingly.

In our study, surgery was the most significant prognosis-influencing factor, which is consistent with the view of previous studies.^[18]^ However, our data show that only 45.3% of the patients underwent surgery, while the number of patients undergoing surgical resection significantly decreased with increasing age. In general, elderly patients tend to suffer from chronic diseases such as hypertension and diabetes, leading surgeons to prefer conservative treatment. Therefore, adjuvant therapy is particularly important for this population. However, the impact of radiotherapy and chemotherapy on the long-term prognosis of perioperative patients is still controversial. Gao et al^[19]^ discovered that compared with surgery alone, cN0 esophageal cancer patients with pathological lymph node-negative or local true lymph node-positive diseases have significant survival benefits from neoadjuvant chemoradiotherapy. In other research,^[9]^ it was found that T3 patients benefit more from surgery plus perioperative chemotherapy, however, perioperative chemotherapy does not present survival benefits to T1-2 patients, and it is an adverse prognostic factor for T1 patients. In this study, we focused not only on those patients who underwent esophagectomy but also on elderly patients who cannot undergo surgery due to various factors such as poor physical condition and oversize tumors. For these patients, chemotherapy (hazard ratio = 0.755; 95%CI: 0.621–0.916; *P* = .004) and radiotherapy (hazard ratio = 0.601; 95%CI: 0.486–0.743; *P* < .001) were significant protective factors.

In previous studies, tumor size was not an independent prognostic factor in patients with node-negative EC.^[6,8,9,19–22]^ However, to our knowledge, tumor size is closely related to the choice of treatment options. In our data, for patients with a tumor size >59 mm, only 20.4% (153/620) of the patients underwent surgery, however, when the tumor size was <22 mm, 69.2% (666/962) of the patients underwent the surgery, which would result in a completely different patient prognosis. In fact, in our univariate and multivariate analysis, the size of the tumor has a significant prognostic significance, we considered that the reason for this result is we used X-tile software to determine the optimal truncation value for the tumor size cutoff point. In contrast, in previous studies, they only classified patients according to their own experience or randomly grouped them, which led to inaccurate conclusions.

So far, some studies have constructed nomograms to predict the long-term prognosis of EC patients,^[23,24]^ but they have not been widely used in clinical practice. However, most studies analyzed only those EC patients who underwent surgery, ignoring elderly patients who cannot tolerate surgery and thus take conservative treatment. In our cohort, only 45.31% (1382/3050) of patients underwent surgery, while 56.95% (1737/3050) and 51.70% (1557/3050) of patients underwent radiotherapy and chemotherapy, respectively. Among patients without surgery, 74.9% (1250/1688) patients received palliative radiotherapy, and 64.0% (1067/1688) patients received palliative chemotherapy, in our opinion, the prognosis of such a population cannot be ignored. Compared with previous studies, this study has several advantages. Firstly, in this study, we used the X-tile to determine the optimal cutoff value for age, and tumor size, which would increase the precision of prognosis prediction, the predictive efficacy of this nomogram was better than that of Zheng et al^[23]^ (0.740 vs 0.708) and Du et al^[24]^ (0.740 vs 0.710). Secondly, due to the particularity of elderly patients, although surgery is the best choice for patients with negative lymph nodes, the prognosis of those elderly patients who cannot be successfully operated on due to poor health still needs to be considered. Finally, a risk stratification system was established to distinguish patients with different prognoses, enabling high-risk patients to have more opportunities to prolong survival and improve their quality of life.

Although this study established a reliable prediction nomogram, some limitations remain. First, we included adjuvant therapy in the establishment of the nomogram, but there was no specific chemotherapy regimen, radiotherapy method, and dose in the SEER database, leading to some bias in the prediction of EC patients.^[25,26]^ Second, there are no certain risk factors affecting the prognosis of esophageal cancer in the SEER database, such as BMI, postoperative complications, nutritional status, targeted drug therapy, or genetic molecule indicators, which contributed to bias. Third, although the SEER database is population-based and the nomogram was internally validated, we did not use external data to confirm the accuracy of the prediction nomogram.

In conclusion, we constructed and validated a nomogram of CSS of node-negative elderly EC patients based on the SEER database, and was well validated, showing higher accuracy than the TNM stage that can be used to identify high-risk patients in clinical practice.

## Author contributions

**Conceptualization:** Yangchen Liu.

**Data curation:** Lianlian Chen.

**Formal analysis:** Lianlian Chen.

**Methodology:** Xinwei Guo.

**Software:** Lang Qin, Xinwei Guo, Faming Yang.

**Supervision:** Xinwei Guo.

**Validation:** Xiaowei Tie.

**Writing – original draft:** Lang Qin.

**Writing – review & editing:** Yangchen Liu.
